# Integrated metagenomic data analysis demonstrates that a loss of diversity in oral microbiota is associated with periodontitis

**DOI:** 10.1186/s12864-016-3254-5

**Published:** 2017-01-25

**Authors:** Dongmei Ai, Ruocheng Huang, Jin Wen, Chao Li, Jiangping Zhu, Li Charlie Xia

**Affiliations:** 1 School of Mathematics and Physics, University of Science and Technology Beijing, 30 Xueyuan Road, Haidian District, Beijing, 100083 People’s Republic of China; 2 Department of Prosthodontics, Ninth People’s Hospital Affiliated with Shanghai Jiao Tong University, School of Medicine, 639 Zhizaoju Road, Shanghai, 200011 China; 3 Oral Bioengineering Lab, Shanghai Research Institute of Stomatology, Ninth People’s Hospital Affiliated with Shanghai Jiao Tong University, School of Medicine, Shanghai Key Laboratory of Stomatology, 639 Zhizaoju Road, Shanghai, 200011 China; 4 Department of Medicine, Stanford University School of Medicine, 269 Campus Dr., Stanford, CA 94305 USA; 5 Department of Statistics, The Wharton School, University of Pennsylvania, 3730 Walnut Street, Philadelphia, PA 19014 USA

## Abstract

**Background:**

Periodontitis is an inflammatory disease affecting the tissues supporting teeth (periodontium). Integrative analysis of metagenomic samples from multiple periodontitis studies is a powerful way to examine microbiota diversity and interactions within host oral cavity.

**Methods:**

A total of 43 subjects were recruited to participate in two previous studies profiling the microbial community of human subgingival plaque samples using shotgun metagenomic sequencing. We integrated metagenomic sequence data from those two studies**,** including six healthy controls, 14 sites representative of stable periodontitis, 16 sites representative of progressing periodontitis, and seven periodontal sites of unknown status. We applied phylogenetic diversity, differential abundance, and network analyses, as well as clustering, to the integrated dataset to compare microbiological community profiles among the different disease states.

**Results:**

We found alpha-diversity, i.e., mean species diversity in sites or habitats at a local scale, to be the single strongest predictor of subjects’ periodontitis status (*P* < 0.011). More specifically, healthy subjects had the highest alpha-diversity, while subjects with stable sites had the lowest alpha-diversity. From these results, we developed an alpha-diversity logistic model-based naive classifier able to perfectly predict the disease status of the seven subjects with unknown periodontal status (not used in training). Phylogenetic profiling resulted in the discovery of nine marker microbes, and these species are able to differentiate between stable and progressing periodontitis, achieving an accuracy of 94.4%. Finally, we found that the reduction of negatively correlated species is a notable signature of disease progression.

**Conclusions:**

Our results consistently show a strong association between the loss of oral microbiota diversity and the progression of periodontitis, suggesting that metagenomics sequencing and phylogenetic profiling are predictive of early periodontitis, leading to potential therapeutic intervention. Our results also support a keystone pathogen-mediated polymicrobial synergy and dysbiosis (PSD) model to explain the etiology of periodontitis. Apart from *P. gingivalis*, we identified three additional keystone species potentially mediating the progression of periodontitis progression based on pathogenic characteristics similar to those of known keystone pathogens.

## Background

Periodontitis results from the hyperimmune response of our body toward pathogenic bacteria resident in the oral cavity, which causes the destruction of periodontal connective tissue [1]. Periodontitis can increase the risk of such systemic conditions as cardiovascular disease, diabetes and obesity [2–4]. According to the latest epidemiological data, more than 47% of U.S. adults suffer from periodontal diseases, including gingivitis and periodontitis [5]. It is generally accepted that the presence of pathogenic bacterial species in host oral cavity, contributes to the onset and development of periodontal diseases. In fact, more than 700 oral microbial phylotypes have already been identified by cultivation, traditional cloning and sequencing [6, 7]. Nonetheless, the exact etiology of periodontal disease, in particular, periodontitis, is yet to be determined.

In earlier years, the etiology of periodontitis was attributed to a few specific plaque species of oral microbiota [8]. For example, using in vitro culture and checkerboard DNA-DNA hybridization, the “red complex” was identified. It consisted of *Porphyromonas gingivalis*, *Treponema denticola* and *Tannerella forsythia*, which are considered to be the most virulent organisms involved in the etiology of periodontitis [9, 10]. Later, Kumar et al. [11] used species-specific 16S rRNA sequencing to expand the catalogue of periodontal pathogens, and the results suggested that periodontitis arises from nonspecific inflammation with diverse progression patterns in response to various plaque species [12]. Then, Marsh et al. proposed that periodontitis is caused by an imbalance of microflora resulting from ecological stress, in turn, enriching the presence of disease-related microorganisms [13, 14].

However, culture-based methods have practical limitations and may overestimate the abundance microbes, resulting in biased estimates. Similarly, species-specific techniques capture only a small fraction of the extremely diverse and complex human oral microbiome. Moreover, neither method can systematically characterize how dental plaque (biofilm) causes destruction of the tooth-supporting structures in the inflammatory state. Recently, the advancement of “omics” technologies has enabled a more holistic approach to the assessment of host oral microbiota. Specifically, it is only with the advent of culture-free, high-throughput sequencing technologies, such as 16S rRNA and shotgun metagenomic sequencing, that we can now comprehensively characterize and compare constituents of bacterial communities with unprecedented resolution. Recent widespread adoption of next-generation sequencing (NGS) technologies has led to even more massive, albeit short, metagenomic datasets [15, 16].

NGS metagenomic sequencing has produced a rich abundance of information about microbial communities compared to traditional sequencing data because of the significant increase in read depth. Previous studies using NGS metagenomic analysis have already advanced our understanding of periodontitis. Based on 16S rRNA and shotgun sequencing, studies like Loreto et al. [17] and Wang et al.[18] have confirmed significant differences in microbial community structures between healthy and periodontally compromised subjects. Orth et al. later used a combination of culture-based methods and high-throughput sequencing to identify a keystone pathogen, *Porphyromonas gingivalis*, which, although prevalent in subgingival samples, can influence host immune response to promote the bacteria that cause periodontitis [19].

As noted above, no consensus has thus far been reached to explain the exact etiology of periodontitis. Therefore, to gain further insight into the composition and structure of oral microbial communities in the context of disease onset, this study first integrated metagenomic sequence data from two previous studies that profiled the microbial community of human subgingival plaque samples, including in total six healthy controls and 37 periodontally diseased samples (among which 14 represent stable periodontitis, 16 represent progressing periodontitis, and the remaining seven samples are diseased but without further classified as stable or progressing. Next, phylogenetic diversity, differential abundance, and network analyses, as well as clustering, were applied to this integrated dataset to compare microbiological community profiles among the different disease states. Accordingly, the paper is organized into three main sections to (1) describe the procedures and software pipeline used for analysis, (2) identify and compare differentially represented microbial species between healthy control and periodontitis subjects, both stable and progressing, using alpha-diversity as the key metric, and (3) cluster species profiles to identify additional keystone species and compare the structure of oral microbial co-occurrence correlation networks using network analysis.

## Methods

### Integration of periodontitis metagenomic datasets

In this study, we first curated and integrated datasets published earlier by Duran-Pinedo et al. and Yost et al. [20, 21], respectively. These studies analyzed gene ontology and phylogenetic composition, as well as catalogued the relevant activities of bacteria in samples with and without periodontitis. However, they did not statistically analyze key factors such as ecological diversity, composition similarity and co-occurrence networks that would have otherwise allowed us to understand the relationship between diversity in the microbial community and the disease state.

This type of study could only be accomplished through the use of a more powerful and integrated comparative metagenomic analysis combining samples from multiple datasets. Owing to high cost, metagenomics projects are typically based on a small number of samples, which limits the power of statistical analysis. Integrating raw data from multiple projects with standardized bioinformatics pipeline would allow us to increase the sample size and boost the statistical power. In this study, by combining data of 13 and 30 samples from two original studies, we arrived at a total of 43 samples, a much larger number and with both healthy and diseased samples. The integrated analysis also allows us to systemically identify the marker and keystone species and exam the co-occurrence networks. Such results were not present in the original studies.

More specifically, we collected all whole genome shotgun sequenced (Illumina sequencing) metagenomic samples from those two studies, which include six controls of metagenomic samples taken from subgingival plaques of healthy individuals, and 37 cases from periodontitis patients. Among the 37 periodontitis metagenomic datasets, 14 samples were from subjects in stable status, as determined by Clinical Attachment Loss (CAL) of < 2 mm compared to their last visit. Sixteen samples were in progressing status, having CAL > 2 mm. Seven samples were from subjects with periodontitis, but their status was unknown. To clarify the terms we used, the disease “state” is either healthy or periodontitis, while the disease “status” can be stable, progressing or unknown.

### Bioinformatics pipeline for integrated metagenomics analysis

**Fig. 1:**
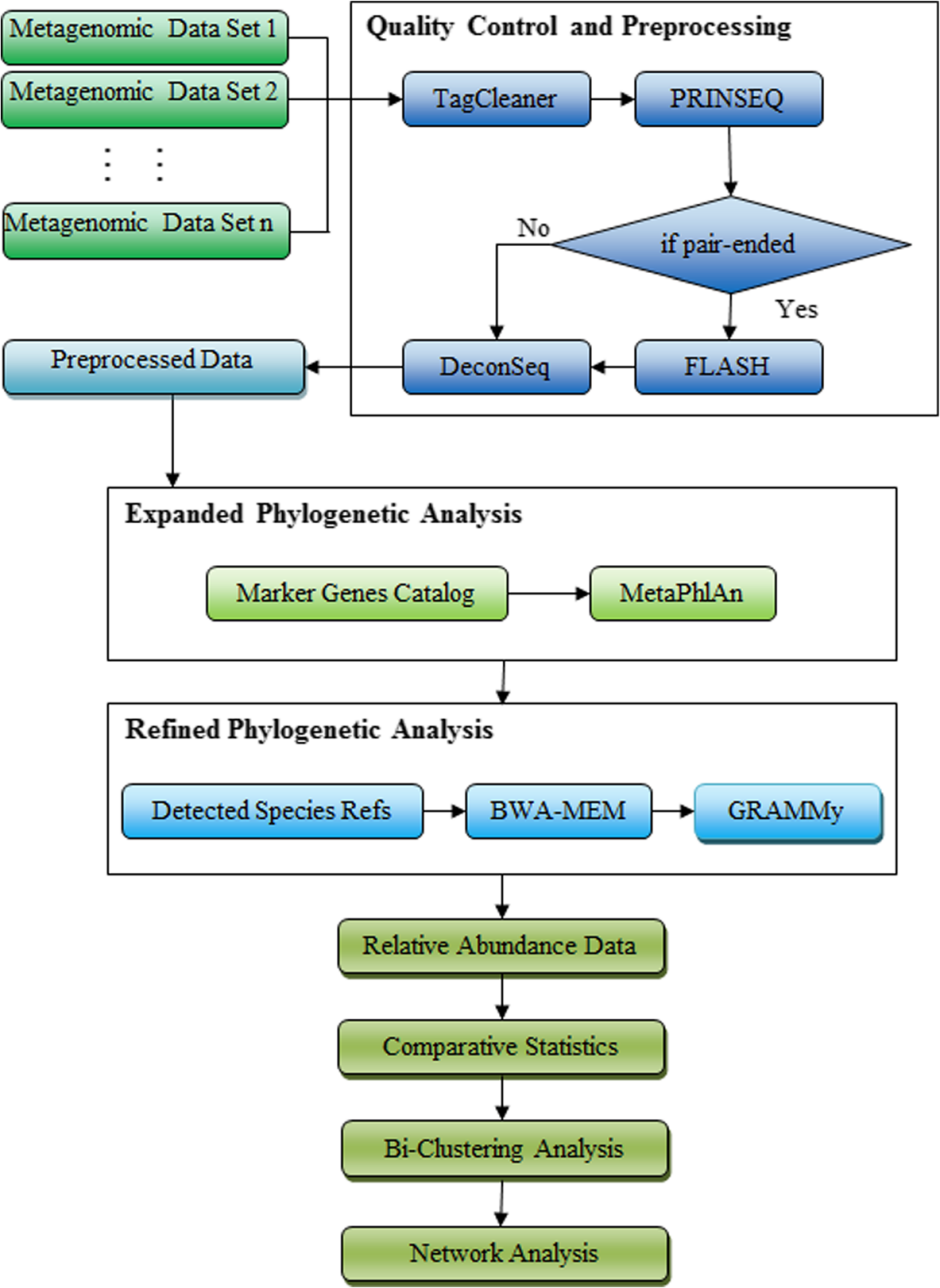
Data preprocessing and bioinformatics pipeline for integrated metagenomics analysis

We constructed a bioinformatics pipeline (Fig. 1) consisting of six steps, as follows: (1) **Quality Control and Preprocessing**, in which TagCleaner, PRINSEQ, Deconseq and FLASH [22–25] were used to remove low quality reads and contamination from the human genome; (2) **Expanded Phylogenetic Analysis**, in which MetaPhlAn [26] was used to sensitively detect the presence of microbial species inoral samples; (3) **Refined Phylogenetic Analysis**, in which GRAMMy [27] was used to accurately estimate the relative abundance of the detected microbial species; (4) **Statistical Analysis**, in which the Dunn test was applied to compare the relative abundance of species and alpha-diversity of microbial communities based on different periodontitis states; (5) **Clustering Analysis**, in which individual oral samples were clustered based on the similarity of marker species abundance profiles; and (6) **Network Analysis**, in which co-occurrence correlation networks based on different periodontitis states were inferred and compared.

### Quality control and preprocessing of metagenomic reads

TagCleaner [22] was used to remove sequencing tags. Tags were predicted by TagCleaner with coverage over 50%. Read sequences at either end representing tags without mismatches were removed. PRINSEQ [23] was then used to remove low-quality reads. Those reads with mean quality score lower than 15, or with a read length out of the range of 3σ from the mean read length, or with more than 1% missing base pairs (bp), were filtered out. Duplicate sequences were also removed. DeconSeq [24] was next used to remove contaminated reads originating from the human genome, i.e., those reads mapped to the human genome with over 98% identity and over 98% base pairs aligned. Finally, FLASH [25] was employed to merge pair-ended reads where paired reads were removed if their overlaps were over 65 bp.

### Expanded phylogenetic analysis

A total of 43 metagenomes sampled from healthy and periodontitis subgingival plaques were analyzed using MetaPhlAn [26], which mapped metagenomic reads to a marker gene catalogue and identified oral microbiota species inhabiting sample environments based on all available reference genomes from the Integrated Microbial Genomes (IMG) system [28]. Expanded phylogenetic analysis allows us to explore the tens of thousands reference species and narrow them down to specific species that are most relevant to our metagenomic samples.

### Refined phylogenetic analysis

GRAMMy [27] was used to estimate the relative abundance of microbes present in the oral sample as identified in the expanded phylogenetic analysis. The complete genomes of present archaea and bacteria, as detected by MetaPhlAn, were downloaded from the Human Oral Microbiome Database [29] to construct the refined reference set for GRAMMy analysis. BWA-MEM [30] was used to align those metagenomic reads that passed the quality filtering to the reference sets. The alignment parameters were set to default, i.e., minimum seed length was set to 19 and mismatch penalty score was set to four, and all plausible alignments were output.

We then applied GRAMMy to the resulting BAM files to estimate oral microbial composition for subgingival plaque samples. GRAMMy was set to default parameters where the e-value threshold was 10e-5, the alignment length threshold was 75 bp, and the identity threshold was 75%. We then used the obtained abundance profiles for the downstream analysis, including, for example, alpha-diversity calculation, statistical testing, biclustering, and network analysis.

### Differential phylogenetic analysis

To identify microbial species differentially present in healthy samples, as well as stable and progressing periodontitis, we applied the Dunn test to compare the relative abundances of detected microbial species (*dunn.test* in the *stats* package of R). We adjusted the Dunn test *p*-values by Benjamini-Hochberg (B-H) correction to control false discovery (*p.adjust* in the *stats* package of R) [31].

### Alpha diversity analysis

We used the Dunn test, as described above, to compare samples from healthy control, as well as stable and progressing periodontitis, relative to differences in microbial community alpha-diversity. We used Shannon index to measure the alpha-diversity of host oral community. Shannon index is defined as,

$$ H={\displaystyle {\sum}_{j=1}^N}\kern0.28em {a}_j\kern0.28em  \log {a}_j, $$

where *N* represents the total number of detected species, and *a*_*j*_ is the relative abundance of the *j-*th species.

In order to test for the potential association between oral microbial community diversity and periodontitis, we performed univariate logistic regression analysis by modeling microbial alpha-diversity as a factor contributing to the probability of developing periodontitis. The model was trained on the six healthy control and 30 periodontal samples whose status, e.g. stable or progressing, were already known. We then used the fitted logistic model as a naïve classifier to predict the potential of developing periodontitis among those remained seven periodontal metagenomic samples whose status was originally unknown and, hence, not part of the fitting data. To run the logistic regression analysis, we used the *glm* function in the *stats* package of R.

### Biclustering analysis

We used the *heatmap.2* function in the *gplots* package of R to bicluster and visually display microbial abundance profiles based on healthy and periodontitis mategenomic samples. In order to generate dendrograms for heatmaps, we applied a *chi*-square transfromation (*decostan* function of *vegan* package in R). The formula is as follows,

$$ {a}_{ij}^{\hbox{'}}=\frac{a_{ij}}{a_i.\sqrt{a{.}_j}} $$

where *a*_*i*_ is the sum over columns (species), which should be one in relative abundance data matrix, and *a*. _*j*_ is the sum over rows (samples). By applying *chi*-square transformation before ordinary biclustering, we can obtain more reasonable distances among metagenomic samples when the data are sparse [32].

We then calculated the Spearman correlations between samples based on differential relative abundances of represented species, using the *cor* function in the *stats* package of R. We converted the correlations to distances by

$$ dist=1-cor $$

and generated the hierarchical clusters of the samples using the *hclust* function *(method=”average”)* in the same R package, which were then automatically converted to dendrograms in the *heatmap.2* function [33]. The “average” method clusters samples by considering the average distance of any member of one cluster to any member of the other cluster.

### Co-occurrence correlation network analysis

Co-occurrence correlation networks can reveal multi-partner microbial interactions [34–38]. To characterize such networks in healthy control, as well as stable and progressing periodontitis samples, we calculated the global Spearman correlations of relative abundances for all pairs of microbial species detected under different states of periodontitis. The *p*-values were adjusted by Benjamini-Hochberg correction. Positive and negative links were drawn between pairs of species whose adjusted *p*-values were less than 0.05. We used the *igraph* package of R to visualize networks under different states of periodontitis.

## Results

### Variability of the most abundant species in periodontitis samples

After preprocessing, healthy samples included an average number of 1,480,414 reads with an average length of 145 bp. Stable samples contained 1,502,809 reads with an average read length of 95 bp, whereas progressing periodontitis samples consisted of an average 746,776 reads and an average read length of 300 bp. The heterogeneity in read length can be attributed to different sequencing run configurations such as 2 *150 and 2 *250 cycles used in the original studies [20, 21]. This sequencing heterogeneity had no effect on our downstream analysis.

From the initial expanded phylogenetic analysis, 135 microbial species were identified by MetaPhlAn. A total of 396 genomes of those species were downloaded from HOMD and used as references for refined phylogenetic analyses. On average, we retrieved three complete genomes for each oral species in the reference set. We used BWA-MEM to map metagenomic reads to references and then used GRAMMy to estimate the relative abundances based on BWA mappings. From healthy and periodontitis metagenomic samples, a total of 70 microbial species were found to have detectable relative abundance by GRAMMy. On average, abundance levels of 47, 31 and 34 microbial species were detected by GRAMMy in subgingival samples from healthy, stable and progressing periodontitis sites, respectively.

**Fig. 2:**
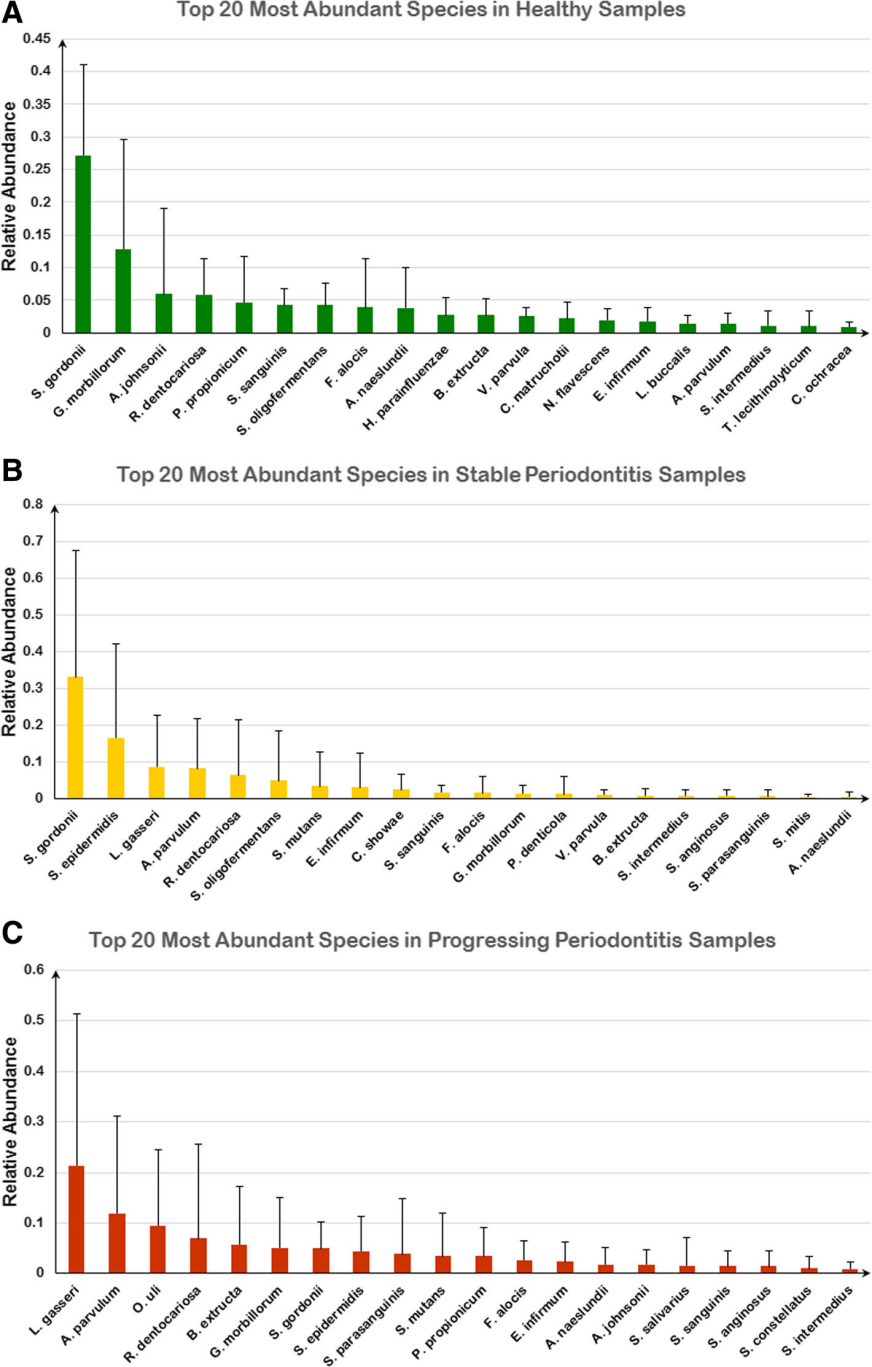
Top 20 most abundant species of human subgingival plaque microbiota. The boxplots of top 20 most averagely abundant microbial species across samples taken from subgingival plaques under different periodontitis states. The same genus is shown in the same color. **a** represents those species in healthy samples, **b**) represents those in stable samples and **c**) represents progressing samples

Figure 2 shows the most abundant microbial species across healthy, stable and progressing subgingival samples. The top ten species in healthy control account for 75.8% (with SD = 11.1%) of total abundance in healthy samples, while total abundance for the top ten species is 87.1% (with SD = 20.9%) for progressing samples and 80.1% (with SD = 18.9%) for stable samples. The proportions of the top ten species in these three groups are significantly different (*P* = 6.61e-10, the *prop.test* function from the *stats* package in *R*). That is the species not in top 10 account for significantly more proportion in healthy samples. In this figure, it can be seen that species from *Streptococcus* and *Rothia* are the most abundant microbes across all healthy, stable and progressing subgingival sites and that they are predominant in the human oral microbiome under both healthy and periodontitis conditions, as expected.

Among other abundant species, periodontitis samples, either stable or progressing, share another three genera, including *Atopobium*, *Lactobacillus* and *Staphylococcus*, while the samples from healthy control and progressing periodontitis oral sites share only one other abundant genus: *Gemella*. On the other hand, samples from healthy and stable periodontitis sites share only *Streptococcus* and *Rothia*. The remaining abundant species specific to healthy samples are from *Actinomyces*, *Filifactor*, *Haemophilus*, and *Propionibacterium*. Of the remaining abundant genera, those specific to progressing periodontitis samples are *Bulleidia* and *Olsenella*, while those specific to stable samples are *Campylobacter* and *Eubacterium*.

It is notable that the abundance distribution of the top ten species is more variable in stable (3 species with SD > +/−15% and average SD = 14.5%) or progressing samples (4 species with SD > +/−15% and average SD of = 13.6%), when compared to healthy control samples (only one species with SD > +/−15% and average SD = 7.9%) (see Fig. 2). In addition, more outliers are found among the top 10 most abundant microbes of stable and progressing samples compared to healthy control samples. Importantly, these observations show a significant reduction of overall ecological diversity in the periodontitis samples, as demonstrated by the concentration of abundance toward only a few dominant species.

### Differentially abundant marker species in periodontitis samples

**Fig. 3:**
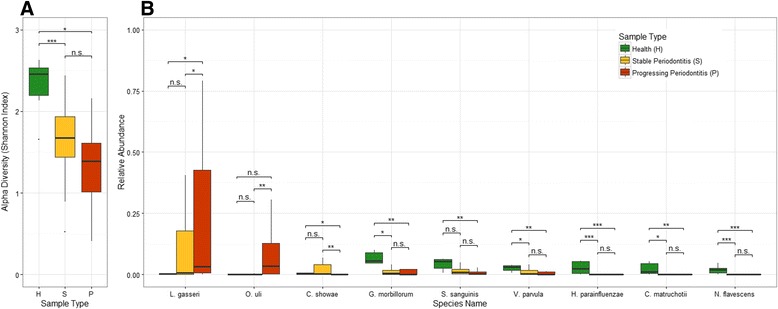
Microbial diversity and abundance difference between healthy and periodontitis samples. The statistical test results of the alpha-diversities and the significantly differentially represented microbial species under different periodontal states. **a** represents box plot and the test results of alpha-diversity, **b**) represents those of the differentially abundant species. As for the box color coding in both subplots, the color of *green* represents healthy samples, *yellow* represents stable samples and *red* represents progressing samples. Statistical significance is coded as: n.s. (*P* > 0.05), *(*P* < =0.05), **(*P* < 0.01), ***(*P* < 0.001) and is labeled above the corresponding boxes

We found nine marker species whose relative abundances were significantly different among healthy (H), stable (S) and progressing (P) periodontitis sites, as shown in Fig. 3b. We found that *Lactobacillus gasseri* (Dunn test, (H vs. P), *P* = 0.014), *Campylobacter showae* (Dunn test, (H vs. P), *P* = 0.034) and *Streptococcus sanguinis* (Dunn test, (H vs. P), *P* = 0.008) were significantly different in progressing periodontitis samples compared to healthy samples. Among them, *Lactobacillus gasseri* was more abundant in progressing samples, while *Campylobacter showae* and *Streptococcus sanguinis* were more abundant in healthy samples.

Five more species had significantly higher relative abundance in healthy samples compared to periodontitis samples, both stable and progressing. Among them, *Gemella morbillorum* (Dunn test, (H vs. S), P =0.010 and (H vs. P), *P* = 0.009) and *Veillonella parvula* (Dunn test, (H vs. S), P =0.028 and (H vs. P), *P* = 0.007) were found in both healthy and periodontitis samples, while *Haemophilus parainfluenzae* (Dunn test, (H vs. S), *P* < 0.001 and (H vs. P), *P* < 0.001), *Corynebacterium matruchotii* (Dunn test, (H vs. S), *P* = 0.016 and (H vs. P), *P* = 0.004) and *Neisseria flavescens* (Dunn test, (H vs. S), *P* < 0.001 and (H vs. P), *P* < 0.001) were only found in healthy samples. The statistical significance of Dunn tests is also shown in Fig. 3b.

The results suggest that they are marker species can be used in biclustering to differentiate among periodontitis states, as discussed later. In addition, *Lactobacillus gasseri* (Dunn test, (P vs. S), *P* = 0.049), *Osenella uli* (Dunn test, (P vs. S), *P* = 0.002), and *Campylobacter showae* (Dunn test, (P vs. S), *P* < 0.001) can differentiate between stable and progressing periodontitis, where the first two species were significantly higher in abundance in progressing periodontitis, and the last species was significantly lower.

### Microbial community alpha-diversity predicts disease status

Alpha-diversity measures the biological diversity of a community, taking both species richness and variance in species proportion into consideration. Using Shannon index as the metric for alpha-diversity, we found the average to be 2.313 for healthy samples, 1.672 for progressing samples, and 1.329 for stable samples. The alpha-diversity of healthy samples is higher than that of progressing samples (Dunn test, *P* = 0.012) and stable samples (Dunn test, *P* < 0.001). However, alpha-diversity of progressing samples is not significantly higher than that of stable samples (Dunn test, *P* = 0.066), which had the lowest alpha-diversity (Fig. 3a).

In order to see if alpha-diversity could be used as a predictor of periodontitis, we fitted a univariate logistic regression model with alpha-diversity as the independent variable and the probability of disease status as the response variable. The fitted values are in Table 1, and the final model is

**Table 1:** The fitted logistic regression model for periodontitis status and Alpha-diversity

	Estimate	Std. Error	zvalue	Pr(>|z|)
Intercept	10.212	3.732	2.736	0.00621
Alpha-diversity	−4.343	1.694	−2.564	0.01035

$$ \log \left(\frac{p}{1-p}\right)=-4.343d+10.212, $$

where *p* represents the probability of an individual having periodontitis, and *d* represents the oral microbial alpha-diversity of the oral microbiome. It can be seen that the coefficient for alpha-diversity in this logistic model is negative, which means that the odds ratio is less than 1. Therefore, the decrease in alpha-diversity of oral microbiome correlates with a higher probability of periodontitis.

The fitted model was then used as a naïve classifier to predict the periodontitis state of seven previously unclassified periodontal samples, which were not used in the fitting. The prediction results, which are found in Table 2, show that six out of the seven subjects were predicted as having periodontitis with high probabilities over 0.7. The remaining subject also had a greater than 50% chance of having periodontitis. If disease status were called as the most probable inference from the model, we would have 100% accuracy.

**Table 2:** Predicted periodontitis probabilities for unknown state patients using the fitted logistic model

Sample	Alpha-diversity	Predicted prob.
Patient 1	2.326	0.528
Patient 2	1.277	0.991
Patient 3	1.606	0.962
Patient 4	2.086	0.760
Patient 5	1.917	0.869
Patient 6	1.375	0.986
Patient 7	1.794	0.919

### Biclustering of community profiles and species in health and periodontitis samples

**Fig. 4:**
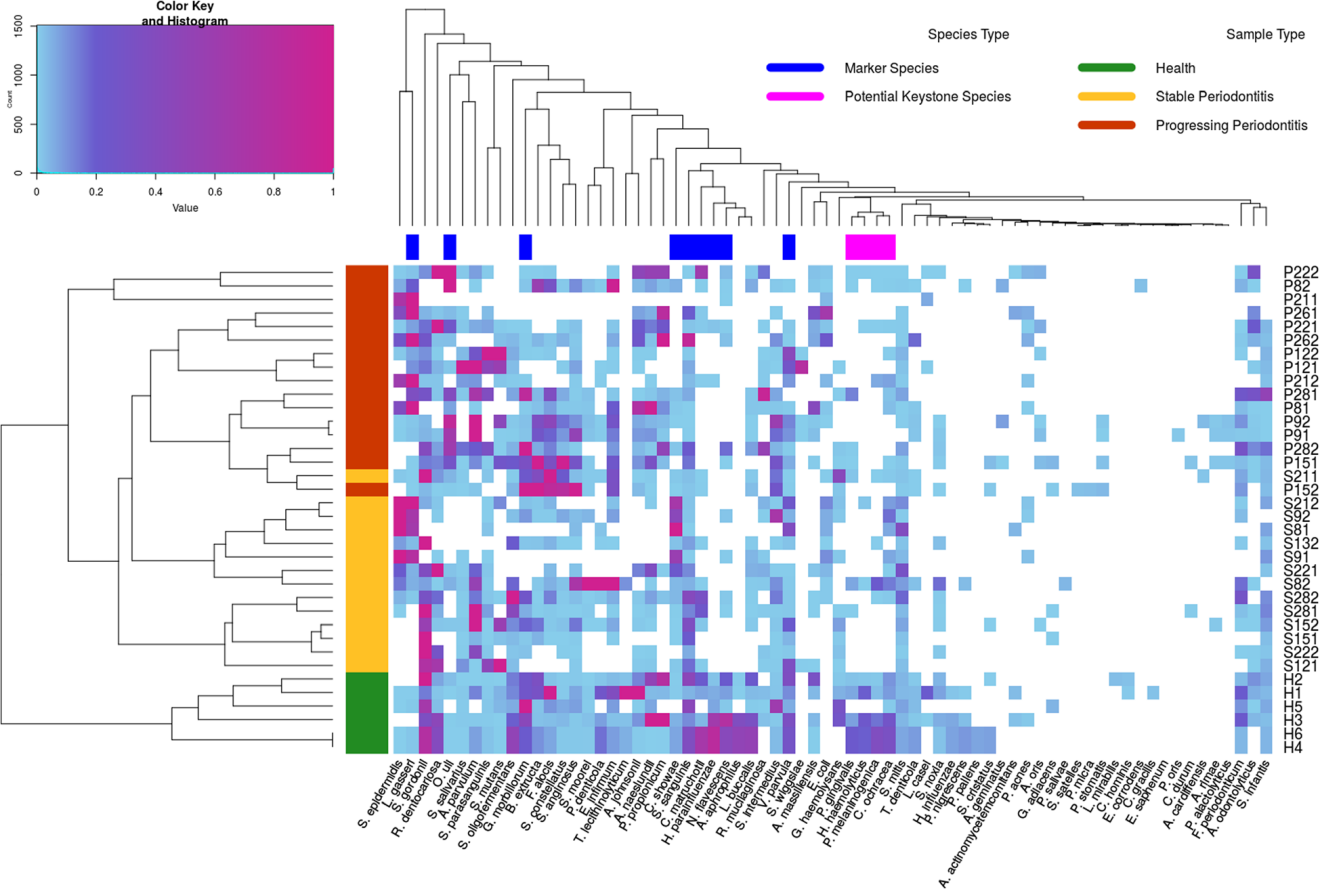
Heatmap and bi-clustering of subgingival samples based on phylogenetic composition of marker species. The heatmap of subgingival samples under different periodontitis states. In the dendrogram on the left side, the color of green represents healthy samples, yellow represents stable samples and red represents progressing samples. The clustering is based on the Spearman correlations of the composition of nine marker species samples, as identified by our analysis

The abundance profiles of 70 microbial species from all samples are shown as a heatmap in Fig. 4. Here, rows are clustered based on Spearman Rank-Order Correlations between the profiles of detected marker species, and columns are clustered for sample abundance similarity between microbial species. We see that all samples from healthy sites are perfectly clustered into one group and that all periodontitis samples are clustered into another group. Moreover, within the periodontitis group, most stable and progressing samples are clustered into subgroups. These results suggest that rank transformed abundance levels are strong predictors of healthy, stable and progressing periodontitis status.

With column clustering, it should be noted that *Porphyromonas gingivalis*, previously known as a keystone pathogen [39], is clustered into a small group with *Haemophilus haemolyticus*, *Prevotella melaninogenica* and *Capnocytophaga ochracea*, indicating that these microbial species have an abundance profile similar to that of *Porphyromonas gingivalis*, thus further suggesting that these species may also play a role as keystone pathogens. The overall distribution by heatmapping intuitively shows these microbial species to be more diverse, i.e., more uniformly distributed, in healthy samples compared to those in stable or progressing samples.

### Patterns of community networks in healthy and periodontitis samples

**Fig. 5:**
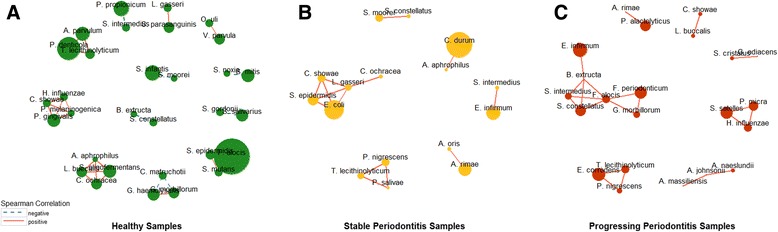
The correlation networks of subgingival species under different peridodontitis states. The co-occurrence correlation networks of subgingival samples under different peridodontitis states. Spearman correlations of relative abundances for all pairs of microbial species were calculated under different states of periodontitis respectively, with *P*-values adjusted by Benjamini-Hochberg correction, and selected those species pairs whose correlation coefficients were over 0.9 and adjusted *P*-values were less than 0.05 as the edges of networks. The size of point represents the average abundance of the species in samples

Finally, we inferred the co-occurrence correlation networks of oral microbial communities inhabiting subgingival plaques under different status of periodontitis based on the *Spearman* correlations of oral species pairs. In the network shown in Fig. 5, all the species pairs with FDR < 0.05 were drawn. They all have a relatively high correlation (correlation absolute value > 0.8). 21 positive (red-colored edges) and seven negative correlations (blue-colored edges) were identified between microbial species in healthy samples. In contrast, only positive correlations were observed in stable (14) and progressing samples (21). Additionally, the total number of correlated species in healthy samples (31 species) was more than that of stable (16 species) and progressing samples (22 species). Subnetworks consisting of more than five correlated microbial species are only found in disease samples, e.g., the subnetwork consisting of five species in stable samples and that of six species in progressing periodontitis samples, respectively.

The five species of subnetwork in stable samples are *Escherichia coli*, *Staphylococcus epidermidis*, *Campylobacter showae*, *Lactobacillus gasseri* and *Capnocytophaga ochracea*. The seven species of subnetwork in progressing samples are *Bulleidia extructa*, *Eubacterium infirmum*, *Fusobacterium periodonticum*, *Filifactor alocis*, *Gemella morbillorum*, *Streptococcus constellatus*, *Streptococcus intermedius*. Two species from the genus of *Streptococcus* are involved in the subnetwork of progressing samples. The overall network structure showed a potential loss of the check-and-balance mechanism through negative feedback in diseased samples.

## Discussion

### Common core microbial species in subgingival plaques

Overall, bacterial communities were found to be very specialized in the subgingival plaque samples. After preprocessing and profiling, an average of 47, 31 and 34 microbial species were detected in healthy, stable and progressing samples, respectively. This indicates a relatively small number of species when compared to all oral microbial phyla. The numbers are consistent with those of previous reports which found as few as 50 predominant species in subgingival plaques, irrespective of health or disease [40, 41]. These results showed that the subgingival plaques sampling procedure was carefully and conservatively performed to avoid possible contamination from the general oral environment. Based on the fact that such number has not substantially changed between their studies and ours, we conclude that the high-throughput, culture-independent methodology faithfully preserves the aboundance structure, even though it is now much more sensitive to the heterogeneity of microbiotas resident in host oral cavity.

Based on phylogenetic analysis (see Fig. 2), we identified such predominant microbial species as *Streptococcus gordonii*, *Streptococcuss anguinis* and *Lactobacillus gasseri*, which are consistent with those identified by Aas et al. and Paster et al. in subgingival samples [40, 41]. Since the oral cavity is the main portal through which most microorganisms enter human bodies, it is possible to detect many transient microbes in the oral environment through metagenomic techniques. Nonetheless, only a few core microbes were consistently found to inhabit subgingival plaques in both this study and those of A as et al. and Paster et al. [40, 41]. These results strongly suggest that periodontitis is induced by inflammatory response to bacterial challenge from the core microbes detected in subgingival biofilm [42]. Thus, the catalogue of these core microbes that persist in subgingival biofilms appears to represent the repertoire of pathogens responsible for disease onset.

In our analysis, we relied on reference genome and read mapping for composition and relative abundance estimation. It is possible that some rare species inhabiting in subgingival plaques were missed out due to low coverage of sampling procedure, low depth of read sequencing, mapping error and other random factors. However, these species’ abundance should be very low even if not truly zero. In this particular study, the microbial species with a relatively high abundance are more likely to be pathogen, because periodontitis is an inflammatory disease that human immune system have active confront with microbes in subgingival plaques. Since our statistics are mainly comparing highly differentiated species, zero abundance levels due to dropouts should not have an effect. In addition, we used standardized bioinformatics pipeline to avoid bias and to estimate the microbial abundance level as accurate as possible. Almost all of the reads got mapped to the provided reference set and therefore there is not much presence of de novo species. That is because human oral microbiota has been extensively studied by clone and culture sequencing in decades, which have generated a very comprehensive set of reference sequences.

### Highly abundant microbial species in subgingival plaques

Among the microbial species discovered in healthy and periodontitis subgingival samples, the genus of *Streptococcus* was found in relative abundance. Many species of *Streptococcus*, such as *Streptococcus gordonii*, *Streptococcus oligofermentans* and *Streptococcuss anguinis*, were among the ten most prevalently abundant microbes at all status of periodontitis. This result suggests that pathogens from the genus *Streptococcus* may be among the most successful early colonizers to clean tooth surfaces in the human mouth by their adherence and metabolic capacities [43]. Based on their predominance in healthy samples, but decrease in periodontitis samples, their colonies might also serve as a source of biofilm adhesion for other colonizers [44].

*Rothia dentocariosa* was also found in high abundance in both healthy and periodontitis samples. In two of the progressing periodontitis samples, it held top abundance rank at 70.4% and 16.8%, respectively, as well as in stable periodontitis samples with relative abundance of 56.2% and 32%, respectively. Although *Rothia* species are often associated with oral health, these results are consistent with previous studies, which found that *Rothia* spp. can reduce oxygen levels around biofilm thus promoting the proliferation of inflammation-triggering anaerobes [17, 45].

Species like *Atopobium parvulum*, *Lactobacillus gasseri*, and *Staphylococcus epidermidis* are highly abundant in stable and progressing subgingival samples, and many of them have already been associated with periodontitis. The *Atopobium* genus, which is high in G + C-content and gram-positive, has previously been identified as prevalent in individuals with periodontitis, but not in healthy subjects. *Lactobacillus* was also found at high percentage in severe periodontitis subgingival samples [46], while *Staphylococcus* genus have only recently been identified as pathogens associated with periodontitis [47]. Our results further strengthen those findings.

*Streptococcus mutans* was also relatively abundant in our subgingival samples. This is particularly interesting because previous observation has shown that this microbial species can create a lactic acid-rich environment, a condition conducive to the development of symbiosis, thus making it possible for other pathogens to thrive [48].

### Prevalent keystone species in healthy and periodontitis subgingival plaques

The keystone species *Porphyromonas gingivalis* previously identified by Orth et al. [19] was found in both healthy and diseased samples, albeit in rather small relative abundance, again hinting that this species may play a role as a keystone pathogen. A keystone pathogen typically remains functional in the background at low abundance, but promotes inflammatory state by altering host immune response to otherwise commensal microbiota. Thus, the existence of *Porphyromonas gingivalis* alone does not cause periodontitis. Rather, if accompanied by proper commensal microbiota, such as species from *Lactobacillus, Staphylococcus* and *Streptococcus* genera [49], in susceptible individuals, *Porphyromonas gingivalis* may be able to evade immune surveillance and mediate the breakdown of normal homeostatic balance in the oral environment [39].

Interestingly, Fig. 4 shows that *Porphyromonas gingivalis* was clustered in a small group with another three species: *Haemophilus haemolyticus*, *Prevotella melaninogenica* and *Capnocytophaga ochracea*. They shared similar patterns with *Porphyromonas gingivalis*, a widely accepted keystone pathogen, in their abundances in healthy and periodontitis samples, which indicates that these three species are likely to play a similar role similar to that of *P. gingivalis* in the oral community by significantly altering the number and community organization of oral commensal bacteria, possibly leading to periodontitis under specific conditions in susceptible bodies. However, more evidence and experimentation are needed to confirm this.

### Differential representation of marker species between healthy and periodontitis subgingival plaques

Marker species are highly differentially represented in healthy and diseased subgingival plaques. As shown in Fig. 3, nine microbial species represent significant difference in relative abundance among the samples studied. In particular, *Lactobacillus gasseri* and *Osenella uli* were found to be significantly more abundant in periodontitis samples. *Campylobacter showae* was observed with significantly higher relative abundance in stable samples. In addition, six microbial species had significantly higher relative abundance in healthy samples compared to periodontitis samples, including *Gemella morbillorum*, *Streptococcus sanguinis* and *Veillonella parvula*, which were observed both in healthy and periodontitis samples, and *Haemophilus parainfluenzae*, *Corynebacterium matruchotii* and *Neisseria flavescens*, which were only detected in healthy samples. The *p*-values of their Dunn tests are shown in the Results section and Fig. 3.

These differentially represented species in subgingival plaques most likely compose the core microbiota directly associated with the etiology of periodontitis. As such, they could be considered markers of periodontitis. As shown in Fig. 4, when we used these nine marker microbial species as representatives of collected samples and clustered the samples based on Spearman correlations of their marker species profiles, the clustering result was nearly the same as their clinical classification (accuracy = 94.4%). This evidence strongly suggests that the combination of differentially abundant species is predictive of periodontitis at different states.

Moreover, unlike pathogen-specific diseases, the etiology of periodontitis is polymicrobial in nature and thus indicative of characteristics of the whole microbial community, or, at least, its core microbiota. It should be noted that the clustering was based on Spearman Rank-Order Correlation, rather than Euclidian distance, which gave us results consistent with clinical classification. This, in turn, means that relative hierarchy of microbial species is highly correlated with progressing periodontitis. Therefore, future studies should look for particular profile patterns of microbial composition that specifically corresponds to different states of periodontitis progression.

### Ecological diversity as a prognostic marker of periodontitis disease

We then focused on the alpha-diversity of samples and its association with the progressing periodontitis. Figure 3 shows a number of microbial species with particularly high outlier abundances in both stable and progressing periodontitis subgingival samples, indicating ecological imbalance among host oral communities. When we compared alpha-diversity of samples between periodontitis and healthy samples, we found that healthy samples had the highest alpha-diversity, while stable periodontitis samples had the lowest with significant difference (see Fig. 3). Furthermore, the fitted univariate logistic model demonstrates the potential value of oral microbial alpha-diversity as a predictor of periodontitis.

### Polymicrobial synergy and dysbiosis (PSD) model of periodontitis etiology

Based on recent metagenomics findings, Orth et al. proposed a Keystone-Pathogen Hypothesis which holds that keystone microbes like *P. gingivalis* may coordinate the onset and progress of periodontitis and these microbial species can alter host-microbial interactions in a manner that interrupts the homeostasis of microbial colonizers and results in inflammatory conditions that set the stage for the development of periodontitis [50]. Our results support this theory in the following way.

According to the PSD model, periodontitis is not caused by a specific microbial species. Instead, inflammation is thought to arise from immune response to the combined activity of core and keystone pathogens. In fact, our phylogenetic profile analysis identified core pathogens and marker species showing significant abundance shifts between healthy and periodontitis samples. For example, marker species *Lactobacillus gasseri, Campylobacter showae* and *Olsenella uli* was more abundant in periodontistis samples, while the marker species *Gemella morbillorum, Veillonella parvula, Haemophilus parainfluenzae, Corynebacterium matruchotii, Neisseria flavescens, Campylobacter showae* and *Streptococcus sanguinis* were significantly more abundant in healthy samples.

**Fig. 6:**
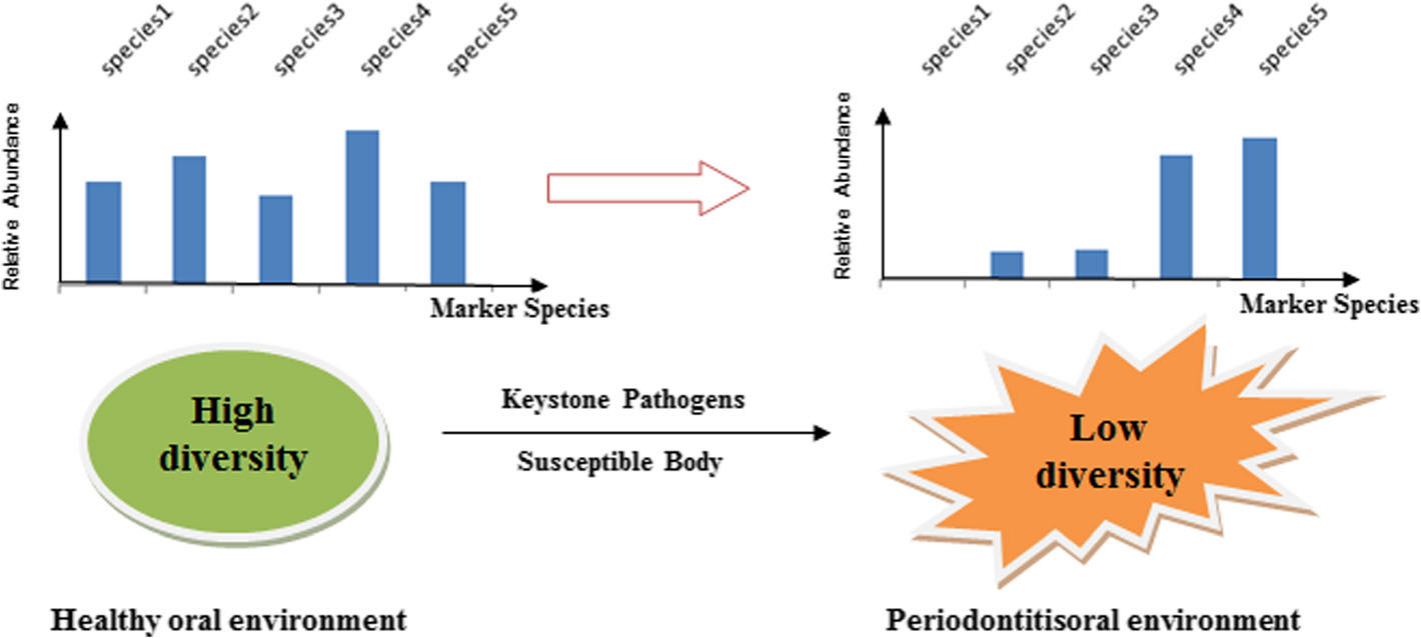
The keystone pathogens mediated polymicrobial synergy and dysbiosis model of periodontitis

We also demonstrated potential keystone species as mediators of this process (see Fig. 6). Based on the PSD model, keystone species gain virulence through cooperating with accessory microorganisms to strengthen the biofilm by creating a powerful synergistic community. Meanwhile, however, previously commensal biofilm species loses homeostasis and drop out from the competition [39, 49, 51]. Indeed, we identified the keystone species *Porphyromonas gingivalis, Haemophilus haemolyticus, Prevotella melaninogenica* and *Capnocytophaga ochracea*, all prevalent at relatively low abundance across all samples and likely acting as mediators of the observed shifts in abundance profiles between periodontitis states [49].

Thus, while these keystone species gain virulence from pathogenic associations with, for example, *Lactobacillus gasseri* and *Osenella uli*, which show relative abundance and thrive in the shifting microenvironment*,* other species lose ground and disappear from the oral environment, such as *Haemophilus parainfluenzae*, *Corynebacterium matruchotii* and *Neisseria flavescens*. Others may decrease significantly in relative abundance, such as *Gemella morbillorum*, *Streptococcus sanguinis* and *Veillonella parvula*. Taken together, we found Shannon diversity for all but one healthy individual to be > 2, similar to the statistics for 10 healthy individuals reported by Bik et al. using 16S RNA sequencing data [52]. On the contrary, almost all periodontitis patients have Shannon diversity < 2 [49]. Importantly, using alpha-diversity as an indicator of microbial flux in a state of dysbiosis, we see a decreasing pattern in periodontitis samples in accordance with the PSD model [51].

The results of our network analysis also support the PSD model and the effects of decreased diversity. The microbial species (nodes) in periodontitis samples have much less interconnectivity (edges), in particular negative associations (blue dashed edges), representing a loss of check-and-balance. There are subnetworks consisting of > 5 correlational microbial species found only in periodontitis samples indicating the pathobionts and keystones thriving through synergistic interaction in the inflammatory state, as suggested in the preceding section. The component species in these subnetworks are different, indicating that the keystones and pathogens active in different states of periodontitis may be different. At this point, the alpha-diversity of subgingival samples has also reached the lowest level.

### Implications of reduced ecological diversity in periodontitis

Irrespective of specific etiology, this and other studies have found oral microbial alpha-diversity to be strongly negatively correlated with periodontitis status [50].

Mutualism and commensalism are hallmarks of diversity among the aggregate of microorganisms resident in human, and such diversity is generally indicative of health [53]. This principle has been tested under conditions of dysbiosis in mucosal diseases of the gastrointestinal tract, such as inflammatory bowel disease and obesity, both considered to be associated with decreased diversity of microbial community [54]. Some researchers, however, have reported that the diversity of oral microbiota is higher in periodontitis compared to healthy subjects [55, 56].

We offered evidence in the present metagenomics study indicating that the principle microbiome diversity is also applicable to periodontal health. Indeed, a healthy oral microbiome is generally reflective of bacterial homeostasis, featuring both synergistic and antagonistic interactions between microbes and between microbes and host [14]. From our results, however, it is likely that polymicrobial synergy eventually leads to dysbiosis by the breakdown of ecological balance in periodontal microbial communities and that the disease state follows.

Accordingly, our results could potentially offer a novel diagnostic and prognostic paradigm based on establishing classification models to distinguish among different states of periodontitis using alpha-diversity and species composition as metrics, together with a sufficient database. However, since the results of the present study cannot pinpoint community diversity as either a cause or result of periodontitis, further study is required to determine whether alpha-diversity of subgingival plaques can, indeed, predict incipient periodontitis in individuals whose clinical symptoms have not yet manifested.

It should be noted that our study was limited in sample size and that we only used average abundances in network construction to reflect the general scope of the oral community in subgingival plaques under different states of periodontitis. However, if longitudinal metagenomics data were to be collected for each periodontitis subject in a larger cohort study, individual oral networks could be constructed, and the underlying correlations among those networks under particular states of periodontitis could be detected. This would provide a better understanding of the etiology of periodontitis.

## Conclusion

This study aimed to discover potential patterns and interactions among microbial communities under three states: healthy control, stable periodontitis, and progressing periodontitis. We integrated metagenomic sequence data of subgingival plaque samples collected from 43 subjects in two previous studies, including Duran-Pinedo et al. [20] and Yost et al. [21]. We found alpha-diversity to be the single strongest predictor of subjects’ periodontitis status (*P* < 0.011) such that healthy subjects had the highest alpha-diversity, while subjects with stable sites had the lowest. We also found that the phylogenetic profiles of nine marker microbes could be applied to differentiate the states of periodontitis to an accuracy of 94.4%. We showed the reduction of negatively correlated species to be another notable signature of disease progression.

Overall, our results support the polymicrobial synergy and dysbiosis model of periodontitis etiology by exploring pathogenic factors that impact microbial homeostasis versus homeostatic imbalance in oral microbiota communities. More importantly, our results consistently show a strong association between the loss of oral microbiota diversity and disease status based on evidence from phylogenetic diversity, clustering and network analyses. This reduction of alpha-diversity is an event distinct from actual etiology. This suggests that metagenomics sequencing can produce phylogenetic profiling results predictive of periodontal health status, possibly leading to novel therapeutic modalities.
